# Aberrant Wound Healing in an Epidermal Interleukin-4 Transgenic Mouse Model of Atopic Dermatitis

**DOI:** 10.1371/journal.pone.0146451

**Published:** 2016-01-11

**Authors:** Yan Zhao, Lei Bao, Lawrence S. Chan, Luisa A. DiPietro, Lin Chen

**Affiliations:** 1 Center for Wound Healing and Tissue Regeneration, College of Dentistry, University of Illinois at Chicago, Chicago, Illinois, United States of America; 2 Departments of Dermatology, College of Medicine, University of Illinois at Chicago, Chicago, Illinois, United States of America; 3 Departments of Immunology and Microbiology, College of Medicine, University of Illinois at Chicago, Chicago, Illinois, United States of America; 4 Medicine Service, Jesse Brown Veterans Affairs Medical Center, Chicago, Illinois, United States of America; NYU Langone Medical Center, UNITED STATES

## Abstract

Wound healing in a pre-existing Th2-dominated skin milieu was assessed by using an epidermal specific interleukin-4 (IL-4) transgenic (Tg) mouse model, which develops a pruritic inflammatory skin condition resembling human atopic dermatitis. Our results demonstrated that IL-4 Tg mice had delayed wound closure and re-epithelialization even though these mice exhibited higher degrees of epithelial cell proliferation. Wounds in IL-4 Tg mice also showed a marked enhancement in expression of inflammatory cytokines/chemokines, elevated infiltration of inflammatory cells including neutrophils, macrophages, CD3+ lymphocytes, and epidermal dendritic T lymphocytes. In addition, these mice exhibited a significantly higher level of angiogenesis as compared to wild type mice. Furthermore, wounds in IL-4 Tg mice presented with larger amounts of granulation tissue, but had less expression and deposition of collagen. Taken together, an inflamed skin condition induced by IL-4 has a pronounced negative influence on the healing process. Understanding more about the pathogenesis of wound healing in a Th2- dominated environment may help investigators explore new potential therapeutic strategies.

## Introduction

Wound healing is a well-regulated complicated process that involves interactions among resident and recruited cells such as epithelial cells, fibroblasts, endothelial cells, inflammatory cells, and interactions of those cells with extracellular matrix molecules, growth factors, cytokines, and chemokines. Inflammatory response is a normal part of the healing process following injury that helps eliminate micro-organisms and injured cells. In addition, Inflammation also assists restoring tissue integrity. However, excessive inflammation may be unfavorable to subsequent healing [1–3].

IL-4 is mainly secreted by Th2 cells, mast cells, eosinophils, and basophils. It was first identified as a factor promoting the growth and differentiation of B lymphocytes [4]. IL-4 is actually a multifunctional cytokine, which has profound effects on not only hematopoietic cells such as B lymphocyte and monocytes/macrophages [5], but also non-hematopoietic cells, such as fibroblasts, where it stimulates the synthesis of extracellular matrix, especially collagens [6–8]. Furthermore, fibrosis in lung and liver has been reportedly associated with the Th2 immune-response, especially the production of cytokines IL-4 and IL-13 [9, 10]. However, there were very limited and conflicting studies investigating the roles of IL-4 in skin wound healing. One study showed that IL-4 was strongly expressed during the early period (days 2–4) of normal wound healing and disappeared after wound closure in a mouse model [11]. This same study reported that topical application of recombinant IL-4 on wounds increased the inflammatory cell infiltrate, but accelerated wound closure while IL-4 anti-sense oligonucleotides significantly inhibited healing [11]. Another study showed that recombinant IL-4 reversed the retardation of wound closure in the bacteria infected acute and chronic wounds accompanied with decreased neutrophil infiltration, but had no effect on closure of acute non-infected wounds [12]. The aim of the present study was to investigate if skin injury in a pre-existing Th2 dominated microenvironment would result in an altered wound healing, such as a chronic wound condition or excessive scar formation. We used a keratin-14 IL-4 epidermal transgenic mouse model [13–22]. This line of mice spontaneously develops a pruritic inflammatory skin condition that resembles human atopic dermatitis, typically demonstrating xerosis, conjunctivitis, chronic skin lesions with T cell, mast cell, macrophage, and eosinophil infiltration, staphylococcus aureus infection and elevation of total serum IgE [13–22]. In the present study, wounds were made on normal looking dorsal skin of IL-4 Tg mice before the onset of skin disease. The results demonstrated that IL-4 Tg mice had significantly impaired wound closure accompanied by drastically elevated levels of inflammation and angiogenesis, as well as decreased deposition of collagen compared to C57BL/6 wild type (WT) mice.

## Materials and Methods

### Animals and wound models

The keratin 14 epidermal IL-4 Tg mouse line was initially established in outbred CByB6 mice in 2001 [15]. It was then backcrossed to C57BL/6 mice for more than 10 generations. The majority of mice develop an atopic dermatitis- like skin condition at the age of approximately 13 weeks [22]. In our current experiments, IL4-Tg mice in a C57BL/6 background were wounded when the mice were eight weeks old and had not yet developed skin disease; C57BL/6 wild type (WT) mice were used as controls. Six 3mm full thickness excisional wounds were made on shaved dorsal skin under ketamine (100mg/Kg) and xylazine (5mg/Kg) anesthesia using a biopsy punch (Acuderm, Inc., Ft. Lauderdale, FL). The wounds were photographed at days 0, 1, 3, 5, 7, 10, 14 and 21 after wounding, and wound sizes were determined by software AxioVision (ZEISS, Oberkochen, Germany). Wound tissues were harvested and stored in either RNAlater (Sigma, St. Louis, MO), embedded in OCT compound or fixed in formalin for future processing. IL-4 Tg mice developing dermatitis during the study were removed from the experiment. Wound breaking strength was examined in another set of experiments. A single 2 centimeter incisional wound was made on the shaved dorsal skin and closed with two surgical clips. The clips were removed at day 7 post-wounding; wounds were harvested at days 14 and 21 to assess wound breaking strength. The University of Illinois at Chicago Institutional Animal Care and Use Committee (IACUC) approved this study. Mice were housed at 22 to 24°C on a 12-h:12-h light/dark cycle and received food and water *ad libitum*. The animals were monitored daily by the animal-care staff and investigators. Any mice displaying signs of infection, severe skin rashes, wasting, and hunching were euthanized. All other mice were sacrificed by lethal CO_2_ overdose followed by cervical dislocation at the end of the experiment. All animal procedures were performed in accordance with the Guide for the Care and Use of Laboratory Animals (National Institutes of Health).

### Indirect immunofluorescence

For immunohistochemical studies, 8μm frozen sections were prepared from wound tissues embedded in OCT compound, air-dried, rehydrated in PBS and fixed in cold acetone for 10 min. Sections were blocked with 10% goat serum for 30 min. Sections were then incubated with either rat anti-mouse Gr-1 (for neutrophil staining, 0.5μg/ml;BD Bioscience, San Jose, CA), rat anti-mouse CD68 (for macrophage staining, 0.5 μg/ml; Abcam, Cambridge, MA), rat anti-mouse CD3 (for T lymphocyte staining, 1.25 μg/ml; SouthernBiotech, Birminham, AL), rat anti-mouse CD31 (for endothelial cell staining, 0.3 μg/ml, BD Biosciences, San Jose, CA), rabbit anti-mouse Ki67 (for proliferating cell staining, 0.5 μg/ml; Abcam), or hamster anti-mouse dendritic epidermal T cells (DETC) (1.25 μ/ml;, BD Biosciences) for 45 min followed by Alexa fluor 488 or 594 goat anti-rat IgG, or goat anti-rabbit IgG Alexa fluor 594, or goat anti-hamster IgG Alexa fluor 594 (Invitrogen, Carlsbad, CA). The staining procedures were performed at room temperature. Stained sections were observed using a fluorescence microscope (Axioskop 40, ZEISS, Oberkochen, Germany) and recorded by a digital camera (AxioCam HRc, ZEISS). CD3 and Gr-1 positively stained cells in the wounds and wound margins were counted and the average number per 20x field was calculated. To quantify the density of CD31-stained blood vessels, the area within the wound bed and CD31-positive area were measured using ImageJ [23]. The vessel density was then expressed as the percent CD31 positive area in the wound bed. Since the staining of CD68 was very intense at some time points in the IL4 Tg mice, it was impossible to accurately count the stained cells. Therefore, CD68 was quantified using the same method for quantification of CD31.

### Hematoxylin/eosin (HE), Masson’s Trichome, and picrosirious red stainings

The rate of re-epithelialization was calculated using the method published previously [24]. Using H&E stained tissue sections, the thickness of epithelium at the wound edge before wound closure or at the center of the wound bed after closure, and the thickness of the wound bed were measured using AxioVision software. Mature and immature collagens were quantified using Image J analysis of the wound bed in picrosirious red stained histologic sections. The percent of mature or immature collagen was calculated as follows: pixels of mature or immature collagen/total pixels of mature and immature collagen x100.

### Real time PCR

Total RNA was extracted from normal skin and wounds of IL-4 Tg and WT mice using TriZol (Invitrogen). One microgram of each RNA sample was treated with DNAse I, and subjected to reverse transcription using a Retro-script kit (Invitrogen). Relative mRNA expression of IL-1β, IL-4, IL-6, IL-10, IL-12, IL-13, IFN-γ, TNF-α, TGF-β1, FGF-7, FGF-10, EGF, VEGF, CCL-2 (MCP-1), CXCL-1 (MIP-2α or KC), CXCL-2 (MIP-2β), lysyl oxidase (LOX), and NLR family pyrin domain containing 3 (NLRP3) was examined using a StepOne Plus real time PCR system (Applied Biosystems, Carlsbad, CA) that employs SYBR Green PCR mix (Roche, Basel,Switzerland) and gene specific primers. All primer sequences for IL-1β, IL-4, IL-6, IL-10, IL-12, IL-13, IFN-γ, TNF-α, TGF-β1, CCL-2 (MCP-1), and GAPDH were as previously described [25]. The primers for CXCL-1, VEGF-A165, NLRP3, MMP-9, and macrophage 2 (M2) marker YM1/Arginase 1(Arg1) were as published in [26], [19], [27], [28] and [29] respectively. The primer sequences of other molecules are listed in Table 1. Levels of mRNA expression in skin of normal WT mice were used as baseline. GAPDH was used as a house-keeping gene for calibration.

**Table 1:** Primer sequences for real time PCR

	Forward (5’-3’)	Reverse (5’-3’)
EGF	CCCAGGCAACGTATCAAAGT	GGTCATACCCAGGAAAGCAA
CXCL-2	CACTCTCAAGGGCGGTCAA	AGGCACATCAGGTACGATCCA
IGF-1	TCATGTCGTCTTCACACCTCTTCT	CCACACACGAACTGAAGAGCAT
LOX	CAAGGGACATCGGACTTCTTAC	TGGCATCAAGCAGGTCATAG
FGF-7	TTTGGAAAGAGCGACGACTT	GGCAGGATCCGTGTCAGTAT
FGF-10	TCAGTGGAAATCGGAGTTGT	TGCTGCCAGTTAAAAGATGC
Collagen I	GGTATGCTTGATCTGTATCTG	AGTCCAGTTCTTCATTGCATT
Collagen III	AGCACCTGTTTCTCCCTT	CTGGTATGAAAGGACACAGAG

### Breaking strength

Breaking strength of 2 cm incisional wounds was examined at days 14 and 21 post-wounding using a tensiometer (Mark-10, Copiague, NY) as described previously [26, 30]. Breaking strength was recorded as weight load (pound, lb) at the point of wound breakage. Two strips from each mouse were tested and the average was used as the wound breaking strength for that individual animal.

### Cell culture

Primary normal human skin epidermal keratinocytes (NHEK, ATCC, Manassas, VA) and human normal dermal fibroblasts (NHDF, PromoCell, Heidelberg Germany) were cultured in 12-well plates to 80–90% confluence and then treated with mitomycin C (10μg/ml, Sigma-Aldrich, St Louis, MO) for 2 hours to prevent cell proliferation. Scratches were produced by application of 3 horizontal and 3 vertical scratch wounds using 0.2ml pipette tips. After washing, the cells were treated with human recombinant IL-4 (50ng/ml, PeproTech, Rocky Hill, NJ), a cocktail of human recombinant IL-1β, IL-4, IL-6, and TNF-α (50ng/ml, PeproTech), or a positive control, human recombinant EGF (50ng/ml, PeproTech) for 24 hours. The defined areas were photographed at 0 and 24 hours post injury, and the numbers of migrated cells in the wounds were counted. For the proliferation assay, 5x10^3^/well NHEK or NHFB cells were cultured in a 96-well plate. After 24 hours, the cells were treated the same way described above and then incubated for 24 hours. A cell proliferation assay was performed using CellTiter 96® Aqueous Non-Radioactive Cell Proliferation Assay kit (Promega, Madison, WI).

### Statistical analyses

Results were expressed as means + standard errors (SEM). A multiple t test or t test was performed using GraphPad Prism (GraphPad Software, San Diego, CA). p values less than 0.05 were considered statistically significant.

## Results

### Wound healing is significantly delayed in the epidermis of IL-4 Tg mice

**Fig 1:**
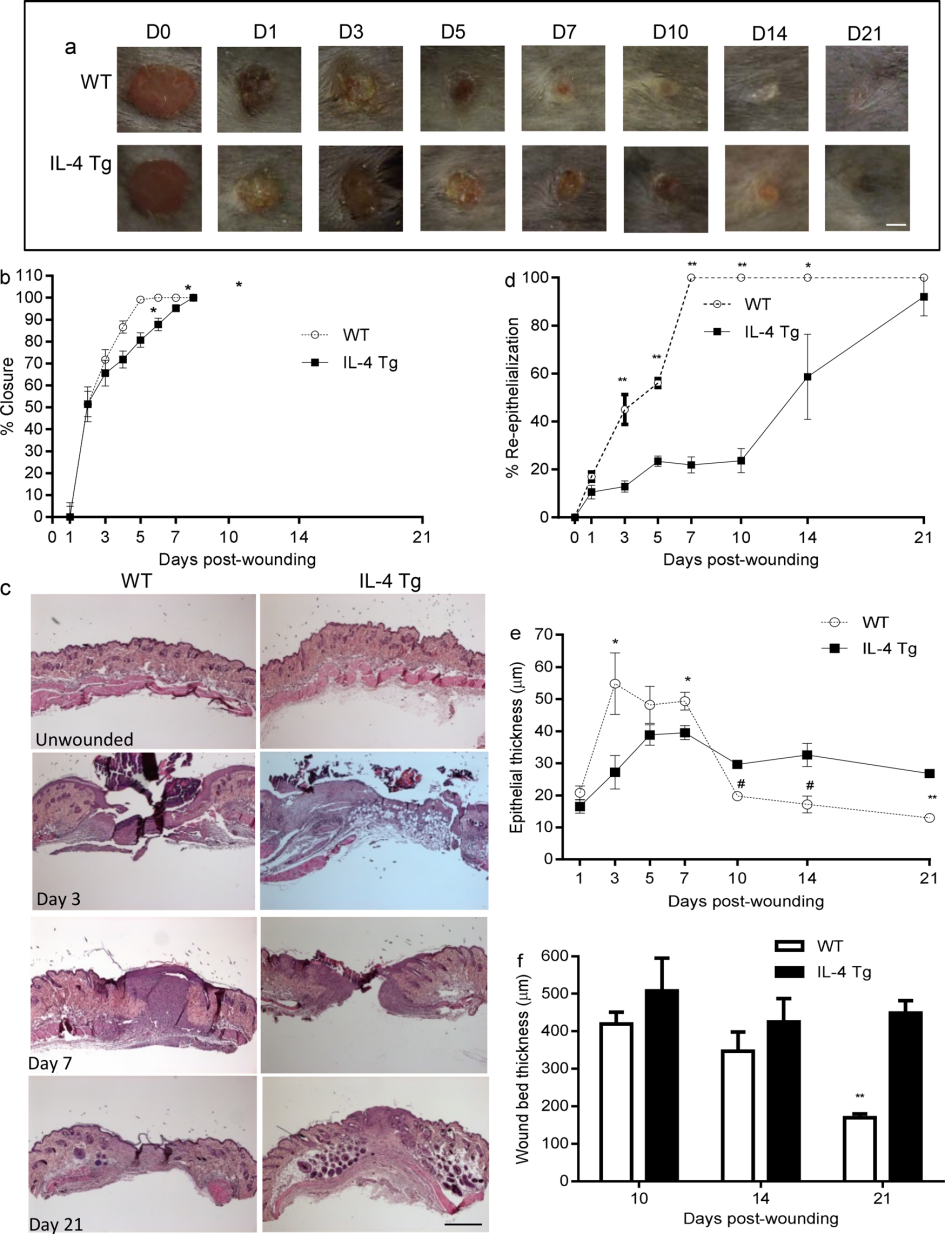
Wound healing is delayed in the epidermis of IL-4 Tg mice. a) Representative photomicrographs of wounds from days 0 to 21 after injury. Six 3mm full thickness excisional wounds were made on the dorsal skin of IL-4 Tg and WT C57BL/j mice. Bar = 3mm. b) Percent of wound closure. Similar results were obtained in another experiment. c) Photomicrographs of HE stained histologic sections of unwounded skin, days 3, 7, and 21 post-wounding. Bar = 200μm. d) Rate of wound re-epithelialization measured by histomorphometric analysis of tissue sections. e & f) Epithelial thickness and wound/scar thickness respectively, based on HE stained sections. * p<0.05, # p<0.01, ** p<0.001 compared to IL-4 Tg mice at the same time point, respectively. The number of mice used at each time point was 5.

IL-4 Tg mice tolerated skin wounding well and all mice survived during the 21 days of study. Three mice developed dermatitis on the dorsal skin in the course of study and were excluded from sample collection. Based on visual observation, wound closure in IL-4 Tg mice was delayed compared to WT mice; this delay was statistically significant at days 5, 7, and 10 (Fig 1A and 1B). Re-epithelialization was examined and quantified in HE stained sections. All wounds were completely re-epithelialized by day 7 in WT mice. In contrast, a significant delay in re-epithelialization was observed in IL-4 Tg mice at days 3, 5, 7, 10, and 14 post-wounding (Fig 1C and 1D). In the IL4-Tg mice, only about 20% of the open wound was covered by new epithelium at day 5 post-wounding, and little additional re-epithialization was seen histologically through day 10. Wound re-epithelialization accelerated after day 10 in IL-4 Tg mice, reaching about 60% and 90% at days 14 and 21 post-wounding e (Fig 1C and 1D). The thickness of the epithelium tip in wounds in WT mice was significantly greater than that of IL-4 Tg mice at day 3 and 7 (Fig 1C and 1E). The pattern reversed from days 10 to 21 (Fig 1C and 1E), at which time the epithelial thickness was greater in IL-4 Tg mice. We did not see marked changes in the histology of unwounded skin in either WT or IL-4 Tg mice (Fig 1C). Overall, the thickness of the wounds and granulation tissue in WT mice was less than that of IL-4 Tg mice, and was significantly different at day 21 (Fig 1F). The results clearly showed that skin wound healing in IL-4 Tg mice was severely impaired, although the wounds eventually healed without signs of infections or excessive scar formation.

### Injury induces higher keratinocyte proliferation and angiogenesis in the epidermis of IL-4 Tg mice than in WT mice

**Fig 2:**
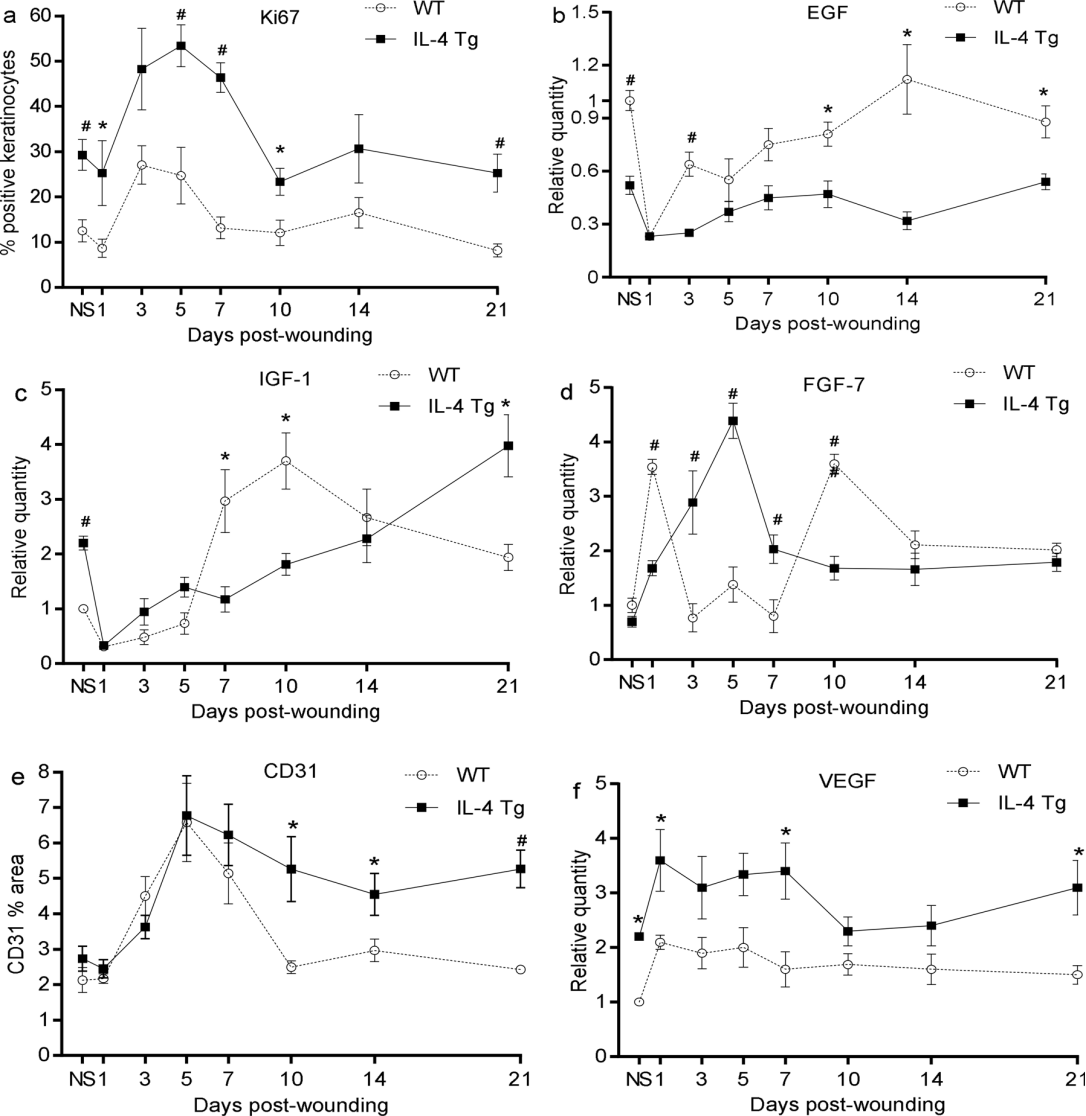
Injury induces stronger keratinocyte proliferation and more angiogenesis in IL-4 Tg mice than in WT mice. Three mm full thickness excisional wounds were made on dorsal skin of IL-4 Tg and WT mice. a) Percent of Ki67 positive keratinocytes. b, c, and d) EGF, IGF-1, and FGF-7 (KGF-1) mRNA expression, respectively. e) Vessel density expressed as the percent CD31 positive area in wounds. f) VEGF mRNA expression. * p<0.05 and ^#^ p<0.01 compared to the other group at the same time point, respectively. NS: normal or unwounded skin. The number of mice used at each time point was 5.

Using Ki67 as a proliferating cell maker, we found that there were significantly more proliferating keratinocytes in both wounds and unwounded skin of IL-4 Tg mice when compared to WT mice (Fig 2A). The number of proliferating keratinocytes peaked at days 3 and 5 for WT and IL-4 Tg mice respectively (Fig 2A). Interestingly, the highly proliferating keratinocytes in IL-4 Tg mice did not migrate efficiently, resulting in delayed re-epithelialization (Fig 1D). In order to study whether the growth factors involved in migration of keratinocyte such as EGF and IGF-1 contributed to delayed re-epithelialization in IL-4 Tg mice, mRNA levels in these factors were examined. We found that EGF and IGF-1 expression were both decreased at day 1 post wounding and then gradually increased thereafter in both WT and IL-4 Tg mice (Fig 2B and 2C). EGF showed a pattern of greater expression in both unwounded and wounded skin of WT mice than in IL-4 Tg mice, especially at days 3 and 10 to 21 after wounding (Fig 2B). Expression levels of IGF-1 at days 7 and 10 were also significantly higher in WT mice than that in IL-4 Tg mice (Fig 2C). Levels of FGF-7 (KGF-1), a critical factor for wound re-epithelialization [31], was also investigated. FGF-7 had two peaks of expression after wounding at day 1 and day 7 in WT mice; levels at these two time points were significantly higher than corresponding levels in IL-4 Tg mice (Fig 2D). In contrast, FGF-7expression exhibited only one peak at day 5 in IL-4 Tg mice (Fig 2D). FGF-7 expression was significantly higher in IL-4 Tg mice than in WT mice at days 3, 5 and 7 (Fig 2D). Similar results were observed for FGF-10 (KGF-2) (data not shown). Blood vessel density in the wounds was evaluated using CD31 staining. There was no significant difference in vessel density between the WT and IL-4 Tg mice in unwounded skin or day 1 through 7 wounds (Fig 2E). The vessel density peaked at day 5 after wounding in both groups (Fig 2E) with similar levels, accounting for about 7% of the total measured areas (Fig 2E). Vessel density decreased slightly in IL-4 Tg mice from days 7 to 21, but was still significantly higher than that in WT (Fig 2E). In contrast, the density in WT mice regressed to the baseline level at day 10 after wounding (Fig 2E). An examination of mRNA levels of the pro-angiogenic factor VEGF showed that VEGF expression in IL-4 Tg mice was higher than in WT mice at all time points, especially in unwounded skin and in wounds at days 1, 7 and 21 post-injury (Fig 2F). The higher VEGF levels in the later stages of healing may explain the slower vascular regression seen in the IL-4 Tg mice. Overall, wounds of IL-4 Tg mice contained more proliferating keratinocytes and a slower vascular regression phase than wounds in WT mice.

### Injury induces more robust inflammatory cell infiltration in wounds of epidermal IL-4 Tg mice than in WT mice

**Fig 3:**
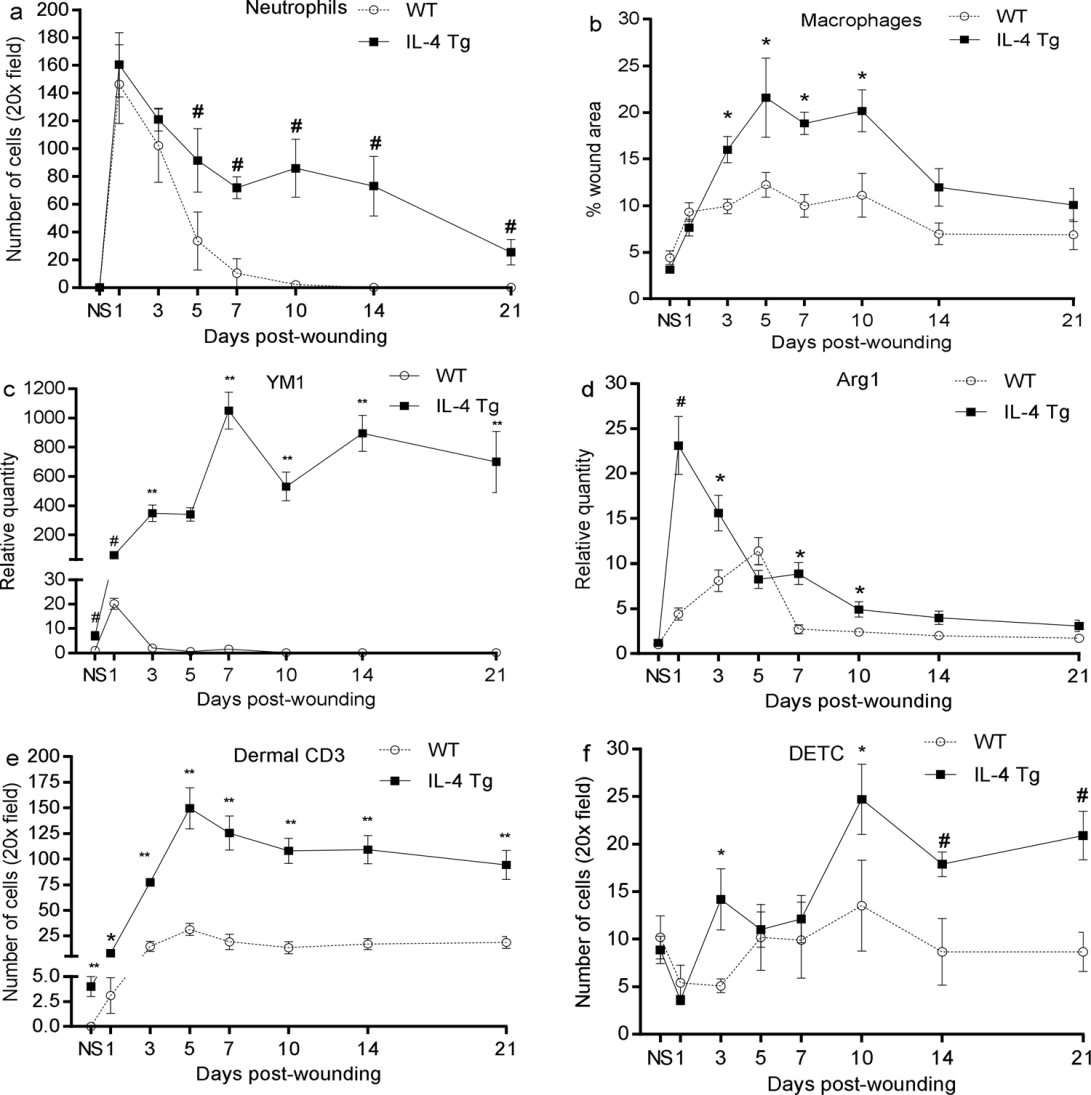
Injury induces robust inflammatory cell infiltration in wounds of IL-4 Tg mice. Three mm full thickness excisional wounds were made on dorsal skin of IL-4 Tg and WT mice. At various time points, frozen sections were prepared for neutrophil (Gr-1), macrophage (CD68), CD3, and DETC staining. Positively stained cells in the wounds and wound margins were counted and the average number per 20x field was calculated. a and b) Time course of the number of neutrophils and macrophages, respectively. c and d) YM1 and Arg1 mRNA expression in wounds respectively. e and f) Time course of the number of dermal CD3+ lymphocytes and DETC, respectively. * p<0.05, # p<0.01, and ** p<0.001compared to WT at the same time point, respectively. NS: normal or unwounded skin. The number of mice used at each time point was 5.

As expected, no neutrophils were present in the unwounded skin of either WT or IL-4 Tg mice (Fig 3A). However, the number of neutrophils sharply increased at day 1 post-wounding, with both strains of mice exhibiting similar levels (Fig 3A). From day 5 to day 21, a significantly higher number of neutrophils were seen in IL-4 Tg mice than in WT mice (Fig 3A). No neutrophils were found in the wounds of WT mice past day 10 post-wounding (Fig 3A). Similar to neutrophil content, there were significantly more macrophages (CD68 + cells) in IL-4 Tg mice than WT mice from day 3 to day 10, with macrophage levels peaking at day 5 in both groups (Fig 3B). mRNA expression of M2 markers in wounds including YM1 and Arg1 was also examined. YM1 expression was significantly higher in IL-4 Tg mice than WT mice, and stayed at an extremely high level over the time course observed (Fig 3C). Arg1 expression in WT mice gradually increased post-wounding, peaking at day 5, and then decreased thereafter (Fig 1D). However, Arg1expression in IL-4 Tg mice was sharply elevated at day 1 post-wounding, then decreased gradually (Fig 1D). Overall, Arg1 expression was significantly higher in wounds of IL-4 Tg than WT mice except at day 5. There were more CD3+ T lymphocytes in IL-4 Tg mice than WT mice as well as in unwounded skin (Fig 3E). CD3+ T cell infiltration was still significantly elevated in IL-4 Tg mice up to day 21 post-wounding (Fig 3E). DETC, a special population of T cells, were also present in greater numbers in IL-4 Tg mice than in WT mice from day 3 to day 21, especially days 3, 10, 14, and 21 post-wounding (Fig 3F). Interestingly, the number of these cells decreased at days 1 and 3 in WT mice and day 1 in IL-4 Tg mice, time points that represent the early phase of wound healing (Fig 3F). The results suggest that injury induces significantly stronger inflammatory cell infiltration in IL-4 Tg mice than in WT mice.

### Injury boosts inflammatory cytokine expression in wounds of epidermal IL-4 Tg mice

**Fig 4:**
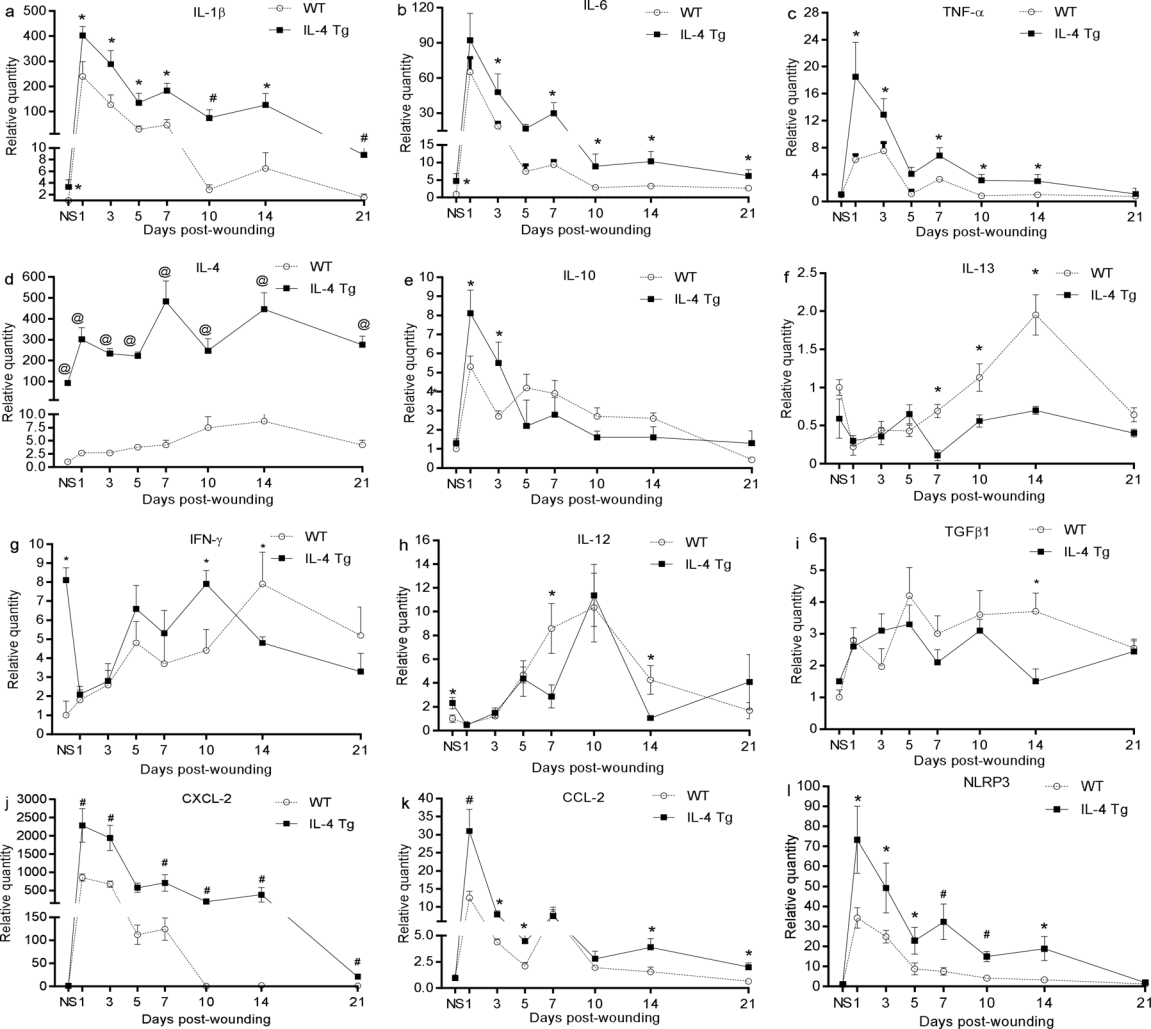
Injury boosts inflammatory cytokine expression in wounds of IL-4 Tg mice. Three mm full thickness excisional wounds were harvested at various time points and total RNA was extracted. After reverse transcription, expression of cytokine mRNAs was semi-quantified using real time PCR: a) IL-1β, b) IL-6, c) TNF-α, d) IL-4, e) IL-10, f) IL-13, g) IFN-γ, h) IL-12, i) TGF-β1, j) CXCL-2, k) CCL-2, l) NLRP3, * p<0.05, ^#^ p<0.01, and @ p<0.001 compared to the other group at the same time point, respectively. NS: normal or unwounded skin. The number of mice used at each time point was 5.

The mRNA baseline level of inflammatory cytokines IL-1β and IL-6, but not TNF-α, was higher in the normal skin of IL-4 Tg mice than that of WT mice (Fig 4A, 4B and 4C). After injury, all three cytokines were expressed at significantly higher levels in wounds of IL-4 Tg mice than in WT mice at nearly all time points (Fig 4A, 4B and 4C). IL-4 expression in wounds of WT mice gradually increased and reached a peak at day 14 (Fig 4D) while, as expected, IL-4 levels in IL-4 Tg mice were hundreds of times greater than in WT mice at all time points (Fig 4D). Other Th2 cytokines, including IL-10 and IL-13, were examined too. Both of these cytokines had similar baseline levels in the two groups, however, IL-10 had significantly higher expression at days 1 and 3 in IL-4 Tg mice than WT mice, while IL-13 had significantly lower expression at days 7, 10, and 14 in IL-4 Tg mice than WT mice (Fig 4E and 4F respectively). The baseline mRNA expression of Th1 cytokines, IFN-γ and IL-12, was also significantly higher in IL-4 Tg mice than that in WT mice (Fig 4G and 4H). Similar levels of TGF-β1 were found in both groups, except that higher expression was observed in WT mice at day 14 (Fig 4I). Since there were more neutrophil and macrophage infiltrates in the wounds of IL-4 Tg mice, expression of CXCL-1 and CXCL-2, two neutrophil chemoattractants, and CCL-2, a macrophage chemoattractant, were examined. We found that CXCL-2 and CCL-2 were significantly increased in wounds of IL-4 Tg mice compared to WT mice (Fig 4J and 4K respectively). Interestingly, no difference of CXCL-1 expression was observed (data not shown). Finally, inflammasome NLRP3 was shown to be more significantly expressed in wounds of IL-4 Tg mice than in WT mice (Fig 4L). The results demonstrated that skin wounds in the epidermis of IL-4 Tg mice had significantly higher expression of inflammatory cytokines and chemokines than WT mice.

### Collagen synthesis/deposition and wound breaking strength are impaired epidermal IL-4 Tg mice

**Fig 5:**
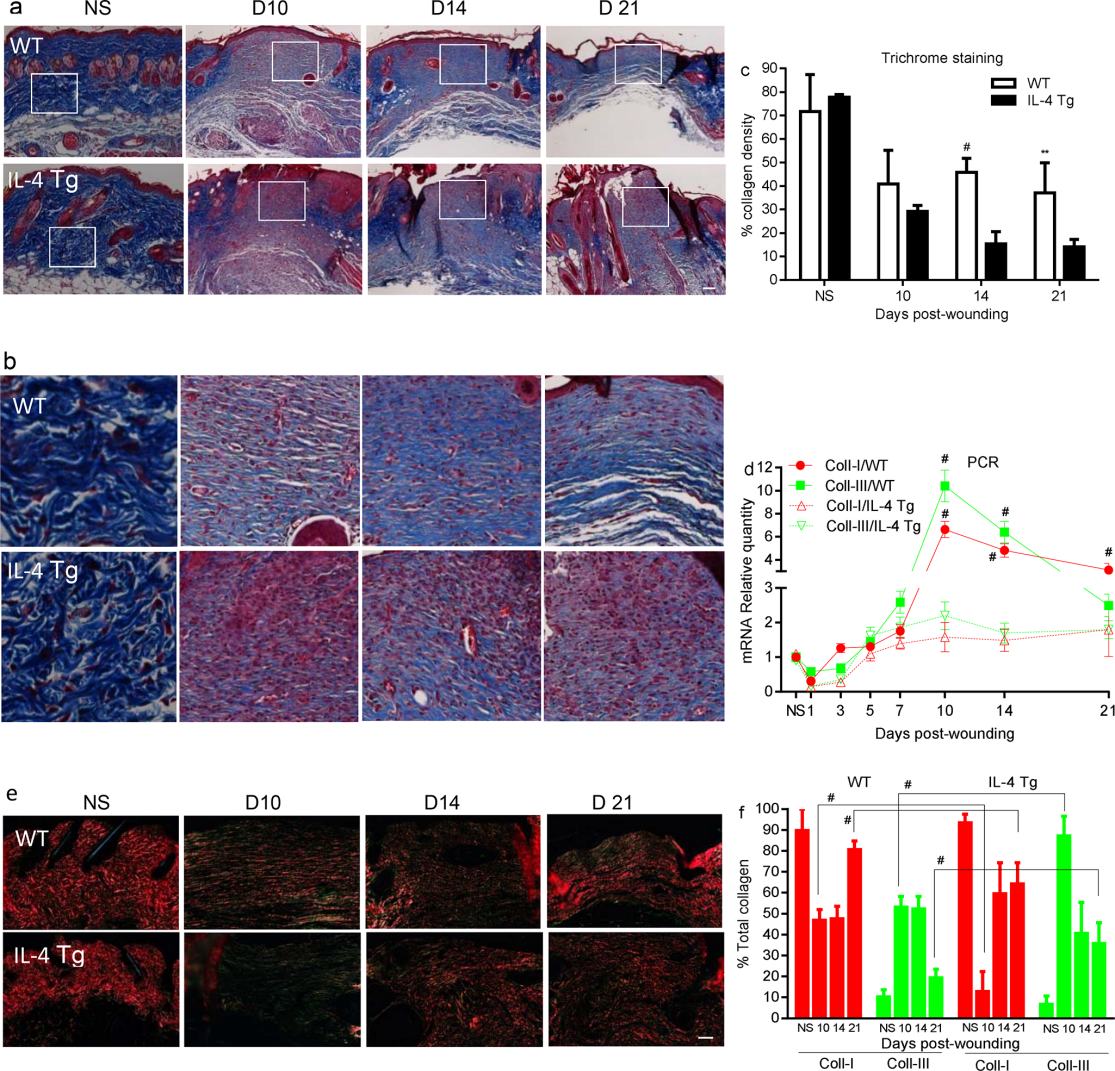
Collagen synthesis and deposition. a) Representative microscopic images of Masson’s Trichrome staining of collagen at days 10, 14 and 21 post-wounding as well as unwounded skin. Blue: stained collagen. b) Corresponding enlarged inserts of a). c) Quantification of blue stained collagen by ImageJ. ^#^ p<0.01 and ** p<0.001 compared to IL-4 Tg mice. d) mRNA expression of collagen I and III. * p<0.05 compared to unwounded skin, ^#^ p<0.01 compared to IL-4 Tg mice. e) Microscopic images of picrosirious red staining of mature (collagen I, red/orange) and immature collagen (collagen III, green). f) Percent of mature and immature collagen based on picrosirious red staining. ^#^ p<0.01 compared to the other group. The number of mice used at each time point was 5.

**Fig 6:**
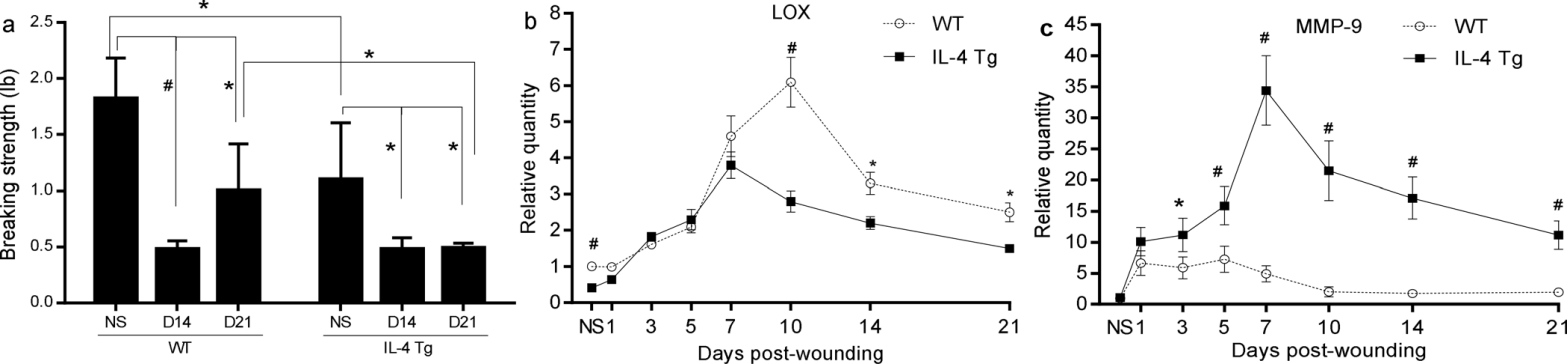
Wound breaking strength. a) Wound breaking strength. * p<0.05, ^#^ p<0.01. b and c) LOX and MMP-9 mRNA expression, respectively. * p<0.05, ^#^ p<0.01 compared to the other group at the same time point. NS: normal or unwounded skin. The number of mice used at each time point was 5.

We next wanted to investigate whether the inflammatory environment induced by the IL-4 transgene affected collagen synthesis and deposition in the wound healing process. Masson’s Trichrome staining revealed there was a significantly higher collagen deposition in WT mice than in IL-4 Tg mice at days 14 and 21 post-wounding (Fig 5A–5C). Analysis if type I and type III collagen mRNA levels showed that there was significantly more expression of collagen I at days 10, 14, and 21 wounds and more collagen III at days 10 and 14 in wounds of WT mice than those of IL-4 Tg mice (Fig 5D). Picrosirious red staining was used to determine the ratio of mature (collagen I) and immature collagen (collagen III) in the healing wounds. Significantly more mature collagen (red/orange) and less immature collagen (green) was observed in day 10 and day 21 wounds of WT mice compared to those of IL-4 Tg mice (Fig 5E and 5F). Both strains showed a shift towards more mature collagen and less immature collagen in day 21 wounds when compared to day 10 wounds (Fig 5E and 5F). Wound breaking strength, a measure of collagen maturity, was significantly lower in IL-4 Tg than WT mice at day 21 (Fig 6A, p<0.05). Interestingly, the breaking strength of unwounded skin in WT mice was significantly greater than that of IL-4 Tg mice (1.8±0.4 lb and 1.1±0.5lb respectively, Fig 6A, p<0.05). We next examined mRNA expression of LOX, an enzyme involving in collagen crosslinking. The expression of LOX in either both unwounded skin and skin wounds at days 10, 14, and 21 of WT mice was significantly higher than that of IL-4 Tg mice (Fig 6B). Furthermore, MMP-9 expression was significantly higher in IL-4 Tg mice than WT mice throughout most of the time course of healing (Fig 6C).

### Effects of IL-4 or cocktail of IL-4 and inflammatory cytokines on migration and proliferation of skin keratinocytes and fibroblasts *in vitro*

**Fig 7:**
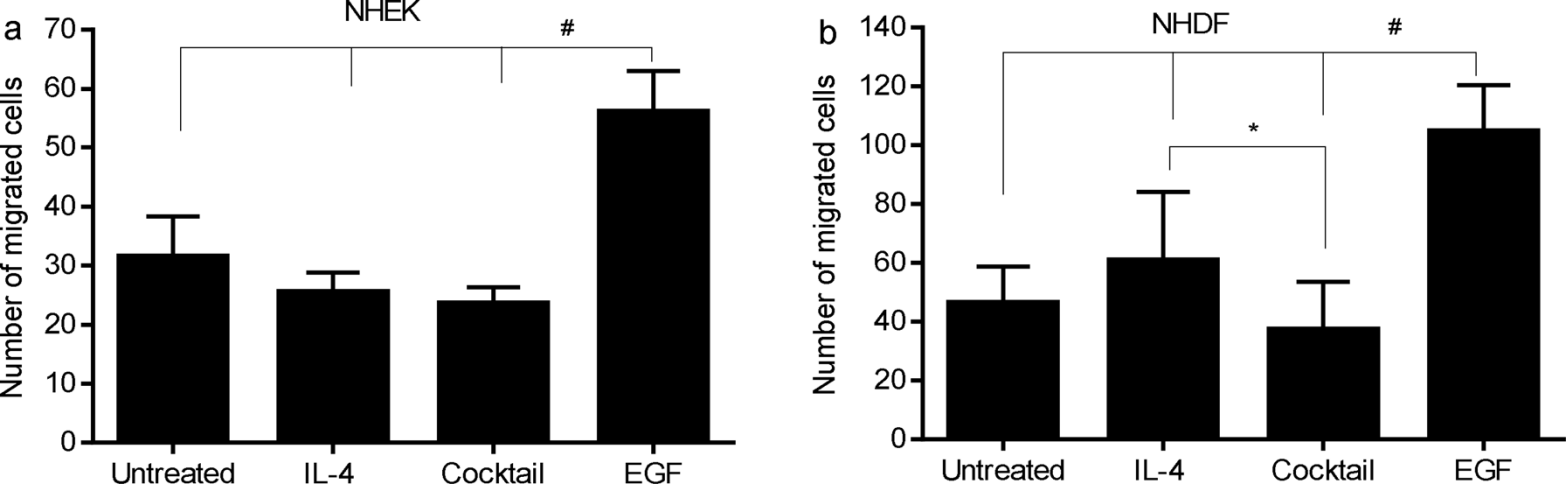
Effect of IL-4 or a cocktail of IL-4 and inflammatory cytokines on migration of skin keratinocytes and fibroblasts in vitro. Scratch wounds were made on monolayers of NHEK and NHDF after mitomycin C treatment to prevent cell proliferation. Cells were then treated with human recombinant IL-4, a cocktail of human recombinant IL-1β, IL-4, IL-6, and TNF-α, or human EGF for 24 hours. The defined areas were photographed at 0 and 24 hours post injury. The numbers of migrated cells in the wounds were counted. Data represent the averages of triplicate wells; similar results were obtained in a replicate experiment. a) NHEK, * p<0.05 between EGF treated group and other groups. b) NHDF, # p<0.001, between EGF treated group and other groups. * p<0.05 between IL-4 and cocktail treated groups.

Even though keratinocytes in the wounds of IL-4 Tg mice had a higher proliferation rate than those in WT mice (Fig 2A), re-epithelialization was significantly slower than that in WT mice (Fig 1D). We examined if IL-4 or combination of IL-4 and inflammatory cytokines would have impact on the migration or proliferation of keratinocytes. Results showed that IL-4 or cocktail of IL-4 and inflammatory cytokines (IL-1β, IL-6 and TNF-α slightly inhibited the migration of human keratinocytes. However, it was not statistically significant (Fig 7A), suggesting that the impairment of re-epithelialization in the IL-4 Tg mice may result from more complicated mechanisms. The migration of human skin fibroblasts treated with the cytokine cocktail was significantly inhibited (Fig 7B) as compared to fibroblasts treated by IL-4 alone, a finding which may partially explain impaired wound healing in IL-4 Tg mice. It seems that IL-4 has greater inhibitory effect on the migration of keratinocytes than on fibroblasts (Fig 7A and 7B). Similar to keratinocytes, these treatments did not have a marked effect on proliferation of fibroblasts (data not shown).

## Discussion

Injury inevitably induces inflammation. However, the extent of the inflammation differs significantly based on the location or tissues involved. Less inflammation may result in optimal healing with minimum scar formation, as described in mucosal or fetal wound healing [32–34]. Excessive inflammation may result in aberrant wound repair, such as delayed healing or chronic wounds [2]. In the current studies, we tried to understand how over-expression of IL-4 in the epidermis affects wound healing using an epidermal IL-4 Tg mouse line. Before onset of atopic dermatitis-like skin disease in these mice, cytokine expression in the skin has already significantly changed compared to WT mice, showing a Th2 dominated profile [20]. Based on the evidence that IL-4 could stimulate fibroblast collagen expression and was associated with fibrosis [9, 10] and the pre-existing inflammation in the skin of IL-4 Tg mice before disease onset, we hypothesized that skin injury might result in a hypertrophic scar or chronic wound. However, injuries on normal looking dorsal skin of these IL-4 Tg mice did not lead to either chronic wounds or hypertrophic scar formation. This result suggests that an underlying inflammatory skin condition does not necessarily lead to an inappropriate healing response. The wounds all closed without noticeable infections. However, wound closure in IL-4 Tg mice was significantly delayed and accompanied by numerous molecular and cellular changes.

In the current study, wounds of IL-4 Tg mice showed robust keratinocyte proliferation throughout the healing time course, yet did also showed delayed wound closure compared to WT mice. This paradox could be explained partially by the lower expression of EGF and IGF-1 in the wounds of IL-4 Tg mice, both factors that enhance migration of keratinocytes [35–39]. Even though IL-4 was highly expressed in the skin of IL-4 Tg mice, in vitro treatment with IL-4 alone did not affect proliferation and migration of keratinocytes. Therefore, the observed changes in proliferation and migration of keratinocytes in the wounds of IL-4 Tg mice are probably regulated by the complicated interactions of various cytokines, chemokines, growth factors, inflammatory cells and other factors.

Inflammatory cells infiltrating into wounds following injury inhibit infection and/or regulate the immune response. Neutrophils are the first line of defense followed by macrophages and then lymphocytes [3]. Significantly increased expression of a neutrophil chemoattractant, CXCL-2, in IL-4 Tg mice is possibly responsible for the increased infiltration of neutrophils seen in the wounds of this strain as compared to WT mice. Neutrophils produce free radicals which not only kill microbes, but also are detrimental to many healthy host cells [40]. Our previous study showed that depletion of neutrophils resulted in accelerated wound healing [41]. Therefore, the significantly increased neutrophil infiltration might contribute to the impaired wound healing in IL-4 Tg mice.

Macrophages in wounds are a critical source of important cytokines, chemokines, and growth factors in wound healing. They play pivotal roles in debridement of the wounded site, cell proliferation, angiogenesis, collagen deposition, and matrix remodeling and are indispensable for successful acute wound healing [3, 40, 42]. In the current study, we found that the infiltration of macrophages significantly increased in IL-4 Tg mice compared to WT mice, which was probably linked to a higher expression of a macrophage chemoattractant, CCL-2 in IL-4 Tg mice. Two major macrophage phenotypes have been identified especially under in vitro conditions; one is a pro-inflammatory macrophage (M1) induced by exposure to IFN-γ and TNF-α, and the other is an anti-inflammatory macrophages (M2) induced mainly by stimulation of IL-4 or IL-13. In the process of tissue repair, M1 dominates the macrophage population in the inflammatory phase while M2dominates in the later stages of proliferation and remodeling [43]. In our study, however, macrophages were markedly increased and the expression of two M2 markers (YM1 and Arg 1) were significantly elevated not only in unwounded skin and early inflammatory stage, but also at the proliferative/remodeling stages in the wounds of IL-4 Tg mice compared to WT mice. The early boost and sustained elevation of M2 in the wound healing process is probably due to the extremely high expression of IL-4 which is the primary inducer of M2. Another possible mechanism is that the inflamed skin or skin wounds of IL-4 Tg mice send a signal (s) to transform newly recruited monocytes/macrophages into M2 phenotype to curb the inflammation in the wounds. Because of the complexity of the cytokines that macrophages are exposed to in wounds, especially in the wounds of IL-4 Tg mice, the phenotypes of macrophages under in vivo conditions are rarely homogeneous, if ever. More investigation is needed to elucidate the roles of these subtypes of macrophages in IL-4 Tg mice.

In the present study, we also observed significant CD3+ lymphocyte infiltration in wounds of IL-4Tg mice throughout the course of the experiment. The functionality of lymphocytes in wounds is currently incompletely understood. Previous reports of the roles of lymphocytes in wound healing are somewhat conflicting. CD4 depletion in rodents demonstrated either no difference in wound healing or impaired healing; CD8 cells have an inhibitory effect on wound healing in terms of collagen deposition and wound breaking strength [44, 45]. Our previous studies showed that lymphocytes isolated from wounds expressed inflammatory and regulatory cytokines such as IL-10, IL-17, IFN-γ, and TGF-β1. However, wound healing in CD4 or CD8 deficient mice occurred normally, with normal wound closure, wound tensile strength, and angiogenesis [26]. These studies suggest that lymphocytes, especially CD4 or CD8 cells, do not play a critical role in acute wound healing. However, given the fact that lymphocytes do express many cytokines in wounds [26], the excess of lymphocytes that infiltrate the wounds of IL-4 Tg mice could impact the wound healing process in a negative way. In addition, our previous studies suggested that lymphocytes isolated from skin draining lymph nodes of IL-4 Tg mice before onset of skin disease spontaneously proliferated without stimulation and proliferated in a larger scale in the presence of mitogens [21]. Similarly isolated CD4 and/or CD8 cells express inflammatory cytokines [20]. These studies indicate that lymphocytes located in the peripheral lymphoid organs directly liked to skin may potentially affect the wound healing too. There are many other disorders accompanied with lymphocyte malfunction which could change the immune response in wound healing, a scenario that needs further investigation. Markedly increased DTECs in IL-4 Tg mice may also partly explain the elevated proliferation of keratinocytes in IL-4 Tg mice, as DETCs produce FGF-7, FGF-10, and IGF-1 and support epithelial proliferation [46, 47].

Collagen synthesis and deposition are critical parts of wound healing which restore and maintain tissue integrity during repair. The incisional wounds of the IL-4 Tg mice had decreased breaking strength when compared to WT mice. This finding relates to the fact that the production of collagen was significantly inhibited in IL-4 Tg mice. In addition, a reduced shift from immature collagen III to mature collagen I, as well as the down regulated LOX expression in IL-4 Tg mice may also contribute to reduced tensile strength. IL-4 is a strong stimulator for collagen synthesis, especially collagen I and III in fibrosis [6–8]. Other inflammatory cytokines including IL-1β and IL-6 can also promote collagen production [48, 49]. These cytokines were drastically elevated in skin wounds of IL-4 Tg mice. In the presence of these potent stimulators, it is not clear why collagen synthesis and deposition were significantly impeded in IL-4 Tg mice. One possible explanation is an increase in factors that degrade collagen. For example, MMP-9, a MMP produced mainly by neutrophils, macrophages, and keratinocytes [50, 51], cleaves gelatin, collagen IV, fibronectin, elastin, laminin, and several other proteins [52]. MMP-9 levels have an inverse correlation with wound closure and wound breaking strength [53, 54]. Therefore, the significantly elevated MMP-9 may contribute to delayed wound healing in IL-4 Tg mice.

Taken together, epidermal over expression of IL-4 results in minor inflammation characterized by increased inflammatory cytokine expression and infiltration of CD3+ lymphocytes in the skin before the onset of an atopic dermatitis-like skin disease. In the context of this Th2 environment, wound closure was significantly delayed and accompanied by vigorous inflammation, epidermal hyper-proliferation, increased angiogenesis, and decreased collagen deposition and tensile strength. Pre-existing inflammation in IL-4 Tg mice changes the complex immune response and has a strong a negative impact on wound healing. IL-4 is the major Th2 cytokine. A Th2 dominated immune response appears in many disorders, including but not limited to, skin diseases such as acute atopic dermatitis [55] and lepromatous leprosy [56] as well as helminth infections [57], allergies asthma [58], and systemic lupus erythematosu [59]. Skin wound healing in these diseases has not been explored. Therefore, the findings in the current study shed new lights on the pathogenesis of wound healing process in a Th2 dominated milieu and may lead to new therapeutic strategies.
