# Correction: Aberrant Wound Healing in an Epidermal Interleukin-4 Transgenic Mouse Model of Atopic Dermatitis

**DOI:** 10.1371/journal.pone.0150443

**Published:** 2016-02-24

**Authors:** Yan Zhao, Lei Bao, Lawrence S. Chan, Luisa A. DiPietro, Lin Chen

In Fig 1, “Wound healing is delayed in the epidermis of IL-4 Tg mice,” panel B appears incorrectly. Please see the corrected Fig 1 below.

**Fig 1:**
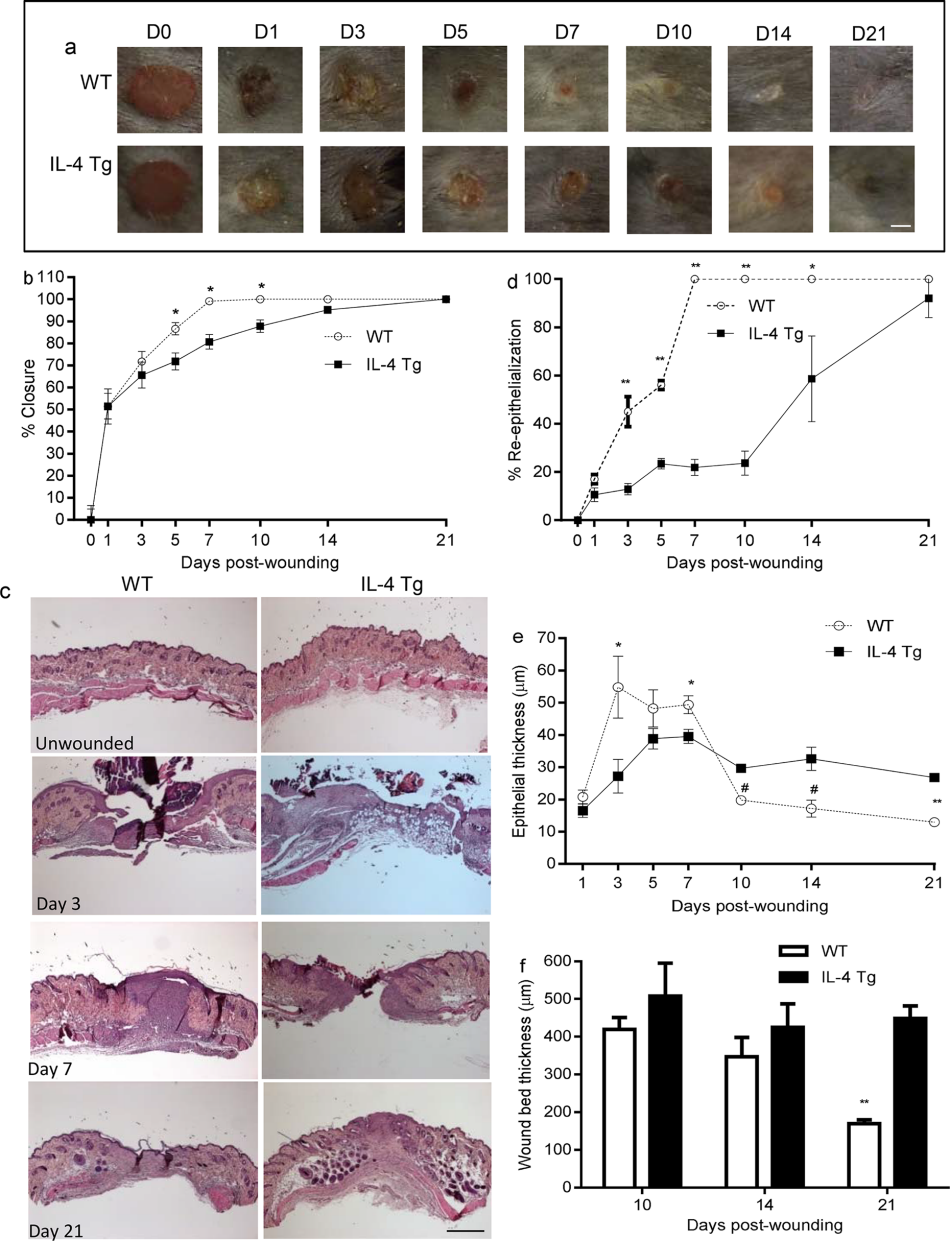
Wound healing is delayed in the epidermis of IL-4 Tg mice. a) Representative photomicrographs of wounds from days 0 to 21 after injury. Six 3mm full thickness excisional wounds were made on the dorsal skin of IL-4 Tg and WT C57BL/j mice. Bar = 3mm. b) Percent of wound closure. Similar results were obtained in another experiment. c) Photomicrographs of HE stained histologic sections of unwounded skin, days 3, 7, and 21 post-wounding. Bar = 200μm. d) Rate of wound re-epithelialization measured by histomorphometric analysis of tissue sections. e & f) Epithelial thickness and wound/scar thickness respectively, based on HE stained sections. * p<0.05, # p<0.01, ** p<0.001 compared to IL-4 Tg mice at the same time point, respectively. The number of mice used at each time point was 5.
